# The estimation of cardiac output by the Nexfin device is of poor reliability for tracking the effects of a fluid challenge

**DOI:** 10.1186/cc11846

**Published:** 2012-10-29

**Authors:** Xavier Monnet, Fabien Picard, Elsa Lidzborski, Malcie Mesnil, Jacques Duranteau, Christian Richard, Jean-Louis Teboul

**Affiliations:** 1 Service de réanimation médicale, Univ Paris-Sud, AP-HP, Hôpitaux universitaires Paris-Sud, Hôpital de Bicêtre, 78, rue du Général Leclerc, 94 270 Le Kremlin-Bicêtre, France; 2 Service de réanimation chirurgicale, AP-HP, Hôpitaux universitaires Paris-Sud, Hôpital de Bicêtre, 78, rue du Général Leclerc, 94 270 Le Kremlin-Bicêtre, France

## Abstract

**Introduction:**

The Nexfin device estimates arterial pressure by the volume clamp method through a finger pneumatic cuff. It also allows to estimate cardiac index (CI_noninv_) by pulse contour analysis of the non-invasive arterial pressure curve. We evaluated the ability of the device to track changes in cardiac index induced by a fluid challenge.

**Methods:**

We included 45 patients for whom a volume expansion (500 mL of saline infused over 30 min) was planned. The volume expansion-induced changes in cardiac index measured by transpulmonary thermodilution (CI_inv_, PiCCO device) and in CI_noninv _were recorded.

**Results:**

In seven patients, the Nexfin could not record the arterial curve due to finger hypoperfusion. Considering both the values obtained before and after volume expansion (n = 76 pairs of measurements), the bias (lower and upper limits of agreement) between CI_inv _and CI_noninv _was 0.2 (-1.8 to 2.2) L/min/m^2^. The mean change in CI_noninv _was 10 ± 11%. The percentage error of CI_noninv _was 57%. The correlation between the changes in CI_inv _and CI_noninv _observed during volume expansion was significant (*P *= 0.0002) with an r^2 ^= 0.31.

**Conclusions:**

The estimation of CI by the Nexfin device in critically ill patients is not reliable, neither for estimating absolute values of CI nor for tracking its changes during volume expansion.

## Introduction

Among the different devices that are available today for estimating cardiac output, the Nexfin technology (BMeye, Amsterdam, The Netherlands) is particularly original. This device provides a non-invasive estimation of cardiac output in two steps. First, this device allows a continuous estimation of the arterial pressure curve through the volume-clamp method [1]. For this purpose, the device includes an inflatable cuff that is wrapped around a finger. It also includes a photoplethysmographic device that measures the diameter of the finger arteries. At each systole, the photoplethysmographic device senses the increase of the finger arteries' diameter. A fast servo-controlled system immediately inflates the cuff in order to keep the arteries' diameter constant. Therefore, cuff pressure reflects the arterial pressure. Its continuous measurement allows estimation of the arterial pressure curve [2]. The second step is to estimate cardiac output from the non-invasive arterial pressure curve. For this purpose, the Nexfin device includes pulse contour analysis software that computes cardiac output from the arterial pressure curve [3].

The Nexfin has been quite largely validated for measuring arterial pressure [4-10]. However, its reliability to measure cardiac index (CI) has been mainly investigated in non-critically ill patients [5,11-15]. Our aim was to assess whether the Nexfin estimation of CI was able to reflect CI in critically ill patients and to track its changes during a fluid challenge.

## Materials and methods

### Patients

This study took place in the medical and surgical intensive care units of a university hospital. It was approved by the institutional review board of our institution (Comité pour la Protection des Personnes Ile-de-France VII). Informed patient (or next-of-kin) consent was obtained from all patients. Patients were prospectively included if they presented an acute circulatory failure for which the attending physician had decided to administer fluid. This decision was based on inadequate tissue perfusion defined by the presence of at least one of the following signs [16-18]: (1) systolic blood pressure <90 mmHg (or a decrease >50 mmHg in previously hypertensive patients) or the need for norepinephrine, (2) urine output <0.5 mL/kg/hr for at least 2 hrs, (3) tachycardia >100 beats/min, (4) skin mottling or (5) blood lactate >2 mmol/L.

### Hemodynamic measurements

All patients had an internal jugular vein catheter and a thermistor-tipped arterial catheter (PV2024 Pulsion Medical Systems, Munich, Germany) in the femoral artery connected to the PiCCO2 device (Pulsion Medical Systems, Munich, Germany) to measure invasive cardiac index (CI_inv_) and global end-diastolic volume (through transpulmonary thermodilution). The femoral arterial line was connected to the pressure sensor PV8115 (Pulsion Medical Systems, Munich, Germany) and the invasive arterial pressure was measured by the PiCCO2 device. In addition, all patients were monitored with a Nexfin device for measuring non-invasive arterial pressure and CI (CI_noninv_). An appropriate size finger cuff was applied around the middle phalanx of the third finger and connected to the device. A height sensor was fixed on one arm at the level of the heart for allowing the device to automatically correct for the hydrostatic pressure influences. If the arterial pressure signal was not obtained from the third finger, another finger was used until a signal could be obtained. If no signal could be obtained from any finger, efforts were made to rewarm the hand before all fingers were tested again.

The Nexfin device continuously records the arterial pressure curve and computes CI_noninv _by pulse contour analysis. This analysis consists in estimating stroke volume by dividing the area under the systolic part of the arterial pressure curve by the aortic impedance [19]. Aortic impedance is determined from a three-element Windkessel model that incorporates the influence of non-linear effects of arterial pressure and of patient's age, height, weight and gender on aortic mechanical properties [20]. The Nexfin method was developed on a database including invasive and non-invasive finger arterial pressures together with thermodilution cardiac output values obtained during cardiac surgery [19,21], in healthy subjects during passive head-up tilt [22] and with low arterial pressure and treatment with catecholamines in severe septic shock [23].

### Study design

At baseline, we measured arterial pressure, heart rate, CI_noninv _and transpulmonary thermodilution variables including CI_inv _and global end-diastolic volume. Immediately after, volume expansion was performed by infusing 500 mL of saline over 30 min. After volume expansion, we again recorded arterial pressure, heart rate, invasive and non-invasive arterial pressure, CI_noninv _and transpulmonary thermodilution variables including CI_inv _and global end-diastolic volume. Patients in whom volume expansion increased CI by more than 15% were defined as 'volume responders' and the remaining ones as 'non-volume responders' [16-18,24].

### Statistical analysis

The normality of data distribution was tested with the Anderson-Darling test. Data are expressed as mean ± standard deviation (SD) or median (interquartile range), as appropriate. CI_inv _was considered as the reference technique [25,26]. Values of invasive vs. non-invasive mean arterial pressure and of CI_inv _vs. CI_noninv _were compared by the Bland-Altman analysis. The percentage error was calculated as two times SD divided by the mean of the reference method [27]. Comparisons of hemodynamic variables between the different study times were assessed using a paired Student *t *test or a Wilcoxon test, as appropriate. Comparisons between volume responders vs. non-volume responders were assessed using a two sample Student *t *test or a Mann-Whitney *U *test, as appropriate. We compared the relative changes of CI_inv _to those of CI_noninv _during volume expansion by linear regression analysis (for percent changes). For assessing the trending ability of CI_noninv_, we constructed a four-quadrant plot, as described by Critchley *et al. *[28]. This allowed calculation of the percentage of total data points for which the directional changes of CI_noninv _(increase or decrease) was concordant with those of CI_inv_. According to the least significant change of CI_inv _[29], we applied a 15% exclusion zone. Correlations were assessed by the Pearson coefficient. A *P *value <0.05 was considered statistically significant. The statistical analysis was performed with the MedCalc8.1.0.0 software (Mariakerke, Belgium).

## Results

### Patients

Forty-five patients were included in the study. Seven patients were excluded because the arterial curve was not obtainable from the Nexfin device, likely due to excessive vasoconstriction. All these seven patients exhibited clinical signs of severe skin hypoperfusion. In these patients, the mean arterial pressure was 43 ± 7 mmHg, cardiac index before the fluid challenge was 2.9 ± 0.5 L/min/m^2 ^and the dose of norepinephrine was 3.8 (2.1 to 6.7) µg/kg/min. On average, the study was conducted 252 (20 to 6520) min after the beginning of the shock episode.

The characteristics of the 38 patients who could be analyzed are summarized in Table 1. Fifty-three percent of patients presented spontaneous breathing activity and 13% presented atrial fibrillation. Volume expansion significantly increased CI_inv _by more than 15% (27 ± 7%) in 16 volume responders (Table 2).

**Table 1:** Characteristics of patients.

Age	65 ± 15
(mean ± SD, years)	
Gender	20/18
(male/female, number of patients)	

SAPS II	55 ± 15
(mean ± SD)	

Acute respiratory distress syndrome	16 (42%)
(number of patients, %)	

Atrial fibrillation	5 (13%)
(number of patients, %)	

Spontaneous breathing activity	20 (53%)
(number of patients, %)	

Tidal volume	6.1 ± 1.3
(mean ± SD, mL/kg of predicted body weight)	

Patients receiving norepinephrine	17 (45%)
(number of patients, %)	

Body temperature	37.8 ± 1.4
(mean ± SD, °C)	

Type of shock	
(number of patients, %)	

Septic	33 (87%)

Hypovolemic	5 (13%)

Dose of norepinephrine	0.4 (0.21-0.60)
(median (interquartile range), µg/kg/min)	

n = 38. SAPS, simplified acute physiology score.

**Table 2:** Hemodynamic changes induced by volume expansion.

		Before volume expansion		After volume expansion	
Heart rate	*volume responders (n = 16)*	104	± 33	110	± 28
	
(mean ± SD, beats/min)	*non-volume responders (n = 22)*	98	± 20	96	± 22
				
Invasive mean arterial pressure	*volume responders (n = 16)*	77	± 16	80	± 20#
	
(mean ± SD, mmHg)	*non-volume responders (n = 22)*	74	± 14	79	± 14
				
Non-invasive mean arterial pressure	*volume responders (n = 16)*	75	± 17	80	± 18#
	
(mean ± SD, mmHg)	*non-volume responders (n = 22)*	70	± 16	76	± 19#
				
Invasive cardiac index	*volume responders (n = 16)*	3.2	± 0.9	3.9	± 1.0#
	
(mean ± SD, L/min/m^2^)	*non-volume responders (n = 22)*	3.8	± 9.0	3.8	± 1.1
				
Non-invasive cardiac index	*volume responders (n = 16)*	3.2	± 1.2	3.6	± 1.1#
	
(mean ± SD, L/min/m^2^)	*non-volume responders (n = 22)*	3.4	± 1.0	3.6	± 1.0
				
Global end-diastolic volume	*volume responders (n = 16)*	726	± 112	767	± 111#
	
(mean ± SD, mL/m^2^)	*non-volume responders (n = 22)*	799	± 137	814	± 156

#*P *<0.05 vs. 'before volume expansion'.

### Non-invasive estimation of mean arterial pressure

**Figure 1:**
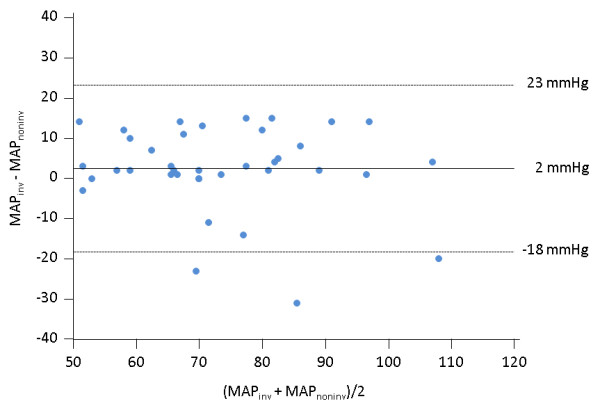
**Bland-Altman plot for the absolute values of mean arterial pressure obtained from the Nexfin device (MAP_noninv_) and from the femoral arterial catheter (MAP_inv_) considering all the pairs of measurements performed during the study**. N = 72; straight line, bias; dashed line, +2D/-2SD limits of agreement.

Considering both the values obtained before and after volume expansion in all patients (n = 76 pairs of measurements), the bias (lower and upper limits of agreement) between the invasive and non-invasive measurements of mean arterial pressure was 2 (-18 to 23) mmHg (Figure 1).

### Non-invasive estimation of cardiac index

**Figure 2:**
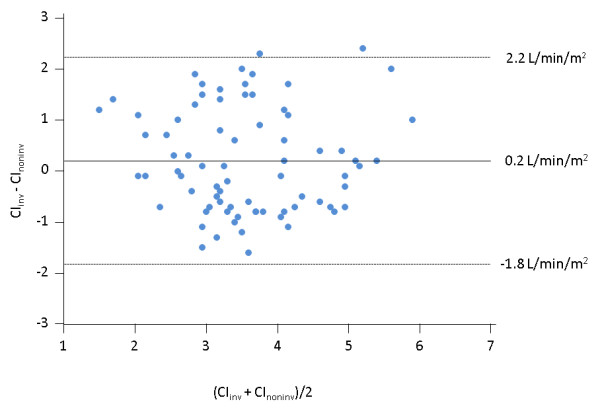
**Bland-Altman plot for the absolute values of cardiac index obtained from the Nexfin device (CI_noninv_) and from transpulmonary thermodilution (CI_inv_) considering all the pairs of measurements performed during the study**. N = 72; straight line, bias; dashed line, +2D/-2SD limits of agreement.

**Figure 3:**
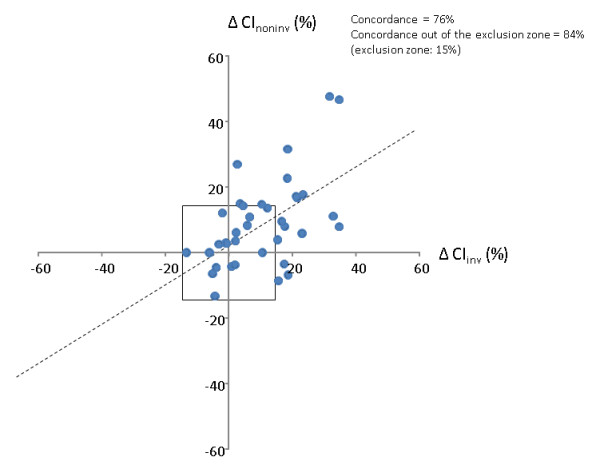
**Trending ability of the cardiac index obtained from the Nexfin device (ΔCI_noninv_) against cardiac index measured by transpulmonary thermodilution (ΔCI_inv_) during volume expansion based on four-quadrant concordance analysis**.

Considering both values obtained before and after volume expansion (n = 76 pairs of measurements), the bias (lower and upper limits of agreement) between CI_inv _and CI_noninv _was 0.2 (-1.8 to 2.2) L/min/m^2 ^(Figure 2). The percentage error of CI_noninv _was 57%. The correlation between the percent changes in CI_inv _and CI_noninv _observed during volume expansion was significant (*P *= 0.0002) with an r^2 ^= 0.31 (Figure 3). The results of Bland-Altman analysis in patients without atrial fibrillation or norepinephrine administration are presented in Table 3.

**Table 3:** Bland-Altman analysis for the comparison between cardiac index measured by the Nexfin device and transpulmonary thermodilution depending upon the absence of atrial fibrillation and norepinephrine infusion.

Subgroup	Bias (L/min/m^2^)	Upper limit of agreement (L/min/m^2^)	Lower limit of agreement (L/min/m^2^)	Percentage error
Whole population (n = 38)	0.2	-1.8	2.2	57%

Patients without atrial fibrillation (n = 33)	0.3	-1.8	2.4	57%

Patients without norepinephrine (n = 21)	0.3	-1.8	2.3	62%

The concordance rate between changes in CI_inv _and CI_noninv _induced by volume expansion was 76% (Figure 3), meaning that in 76% of instances, CI_inv _and CI_noninv _changed in the same direction. When excluding changes lower than 15%, the concordance rate was 84% (Figure 3).

An increase in CI_noninv _≥15% allowed to detect volume responsiveness, that is, an increase in CI_inv _≥15%, with a sensitivity of 43% (95% confidence interval: 20 to 70%), a specificity of 95% (77 to 100%), a positive predictive value of 88% (44 to 100%) and a negative predictive value of 70% (51 to 85%).

## Discussion

The main finding of this study was that the non-invasive estimation of cardiac output provided by the Nexfin method was not reliable compared to transpulmonary thermodilution in critically ill patients even though, during volume expansion, CI_noninv _and CI_noninv _often changed in the same direction.

In the recent years, many efforts have been made to develop new techniques for monitoring cardiac output, with the particular aim of reducing their invasiveness. In this regard, the Nexfin technique seems particularly seductive since it only requires a finger pneumatic cuff. Nevertheless in the present study, we found that the Nexfin device was not reliable to estimate the absolute value of cardiac output. The Bland-Altman analysis showed a wide range for limits of agreement and the percentage error, which must be below 30% if transpulmonary thermodilution is used as the reference technique [27,29], was much higher. More than in a given value of cardiac output, clinicians might be more interested in cardiac output changes. In this regard also, the Nexfin estimation of cardiac output was not reliable. Indeed, the correlation between CI_noninv _and CI_inv _was only weak. If analyzing this trending ability by a four-quadrant approach, as proposed by Critchley *et al. *[28], we found that the concordance rate was 84% when excluding from analysis changes in cardiac output that were too small to have any technological significance. It means that, in a majority of cases, CI_inv _and CI_noninv _changed in the same direction, which would suggest a good trending ability of CI_noninv_. Nonetheless, this four-quadrant analysis only takes into account the direction of cardiac output changes and not their magnitude, that is, it only allows a rough evaluation. For clinical practice, not only the direction but also the amplitude of CI changes might be important. For instance, the response to a fluid challenge is assessed by precisely measuring the relative change in CI that it induces. In this regard, we report a poor ability of CI_noninv _to detect a positive response to fluid administration, in particular with a low sensitivity.

This poor reliability of the Nexfin device to estimate cardiac output is in discrepancy with some previous results obtained with the same technique [5,11-15,30]. A recent publication also reported a good ability of the Nexfin device to estimate cardiac output measured by transpulmonary thermodilution as well as its changes observed over time or during a passive leg-raising test [6]. The most plausible hypothesis explaining the discrepancy of such previous results with our findings is the difference in the population of interest. The studies that demonstrated a better reliability of the Nexfin were conducted in the operating theatre [11,12], in cardiac surgery patients after discontinuation of mechanical ventilation and inotropes [5], in patients undergoing resynchronization therapy [14], in an echocardiography laboratory [13] or in healthy subjects [15]. By contrast, in the present study, we included critically ill patients, a majority suffering from septic shock. Interestingly, our results are in agreement with another study reporting a poor reliability of the Nexfin and that was also conducted in intensive care unit patients [31]. In fact, such patients are much more likely to have an impaired finger perfusion, due either to endogenous sympathetic stimulation, to norepinephrine administration or to sepsis-related microcirculatory abnormalities. In this regard, the fact that the reliability of the Nexfin device was not different between patients with and without norepinephrine suggests that exogenous amines administered might not be the only factor explaining a poor finger perfusion in such critically ill patients. Poor finger perfusion very likely impedes a correct assessment of the finger pressure curve by the volume-clamp method, which includes an analysis of the finger photoplethysmographic signal. In this regard, we observed that the non-invasive arterial pressure curve was not obtainable in a relatively large proportion of our patients. We made a similar observation with another volume-clamp device in a similar population [32]. This suggests that such a limitation is inherent to all these techniques of non-invasive arterial pressure assessment.

With the Nexfin device in the present study, we could not determine whether the poor reliability of estimation of cardiac output was related to a poor assessment of the arterial pressure curve or to a failure of the pulse contour analysis process since this proprietary process was unknown to us. Nevertheless, we hypothesize that the pulse contour analysis did not account a lot for the results. Indeed, the therapeutic intervention we used, that is, volume expansion, is known to alter the pulse contour analysis to a lesser extent than treatments modifying the properties of the arterial tree, such as vasoactive agents [33-35].

The estimation of arterial pressure by the Nexfin technique is based on the old volume-clamp method [1] but also includes an individual calibration technique (Physiocal) developed by Wesseling *et al. *[36]. Compared with the previous Finapress device, the Nexfin device has benefited from many technological improvements [7]. It has been reported to reliably estimate arterial pressure [4-10]. In the present study, the assessment of such reliability was based only on the comparison of mean arterial pressures provided by the Nexfin vs. the arterial line. Indeed, due to the pulse wave amplification phenomenon, the systolic and diastolic values of arterial pressure are physiologically different between the brachial pressure, estimated by the Nexfin device, and the femoral pressure that we invasively measured. By contrast, the mean arterial pressure is almost constant along the arterial tree and we used it for assessing the reliability of the Nexfin arterial pressure estimation. The bias between non-invasive and invasive mean arterial pressures was low, while the limits of agreement were relatively high. Previous studies reported better results for this device [4-10]. Like for the estimation of cardiac output, these different results might be explained by the fact that we included patients with septic shock receiving vasopressors, in whom the analysis of the non-invasive arterial pressure curve might be difficult due to a poor finger perfusion. The fact that the Nexfin device was of poorer reliability in critically ill patients than reported in the perioperative setting [5,11,12] suggests that such a device is more suitable for the operating theatre than for intensive care units.

A first limitation of our study is that we could not investigate the ability of the Nexfin device to estimate pulse pressure variation, as we did for a similar device in a previous study [32]. This was due to the fact that we included a majority of patients in whom pulse pressure variation is of difficult measurement and interpretation, that is, spontaneous breathing activity and/or cardiac arrhythmias and/or acute respiratory distress syndrome [37,38]. Also, although the manufacturer recommends that the finger cuff should be applied to the middle phalanx of the second or third fingers, it was not the case in all the patients of the study since the position of the cuff was changed when the arterial pressure could not be obtained from the third finger. In addition, we cannot exclude that when the patient's hand was rewarmed, this could have distorted the Nexfin measurement by changing the microcirculation. Finally, although we hypothesize that the discrepancy between the present study and previous publications was related to the type of included patients, we did not directly compare the device in similar conditions between critically ill and perioperative patients. Such a comparison remains to be performed.

## Conclusions

The estimation of cardiac output by the Nexfin device in critically ill patients is not reliable, neither for estimating absolute values of cardiac output nor for tracking its changes during volume expansion.

## Key messages

• The estimation of cardiac index by the volume-clamp method in critically ill patients was not reliable.

• The method could not reliably track the changes in cardiac index induced by volume expansion.

• Due to finger hypoperfusion, the technique could not estimate cardiac index in a relatively large proportion of these patients with an acute circulatory failure.

## Abbreviations

**CI_inv_**: cardiac index measured invasively by transpulmonary thermodilution; **CI_noninv_**: cardiac index measured non-invasively by the Nexfin device.

## Competing interests

Profs Jean-Louis Teboul and Xavier Monnet are members of the Medical Advisory Board of Pulsion Medical Systems. The other authors have no financial interest to disclose.

## Authors' contributions

XM conceived the study, performed analysis and interpretation of the data and drafted the manuscript. FP performed the collection of data, contributed to analysis and interpretation of the data and to drafting of the manuscript. EL performed the collection of data, contributed to analysis and interpretation of the data and to drafting of the manuscript. MM performed the collection of data, contributed to analysis and interpretation of the data and to drafting of the manuscript. JD conceived the study, participated in its design, contributed to analysis and interpretation of the data and helped to draft the manuscript. CR participated in the design of the study, contributed to analysis and interpretation of the data and helped to draft the manuscript. J-LT conceived the study, participated in its design, contributed to analysis and interpretation of the data and helped to draft the manuscript. All authors read and approved the final manuscript.
